# From Nanocrystals and Nanocomposites to Microcrystals: The Role of Simonkolleite/ZnO in Overcoming Bacterial Resistance and Ensuring Biocompatibility

**DOI:** 10.1021/acsomega.4c09594

**Published:** 2025-05-15

**Authors:** Jerusa M. de Oliveira, Maria P. C. Costa, Hugo F. Perini, Rafael O. Trevisan, Larissa I. M. de Almeida, Samanta L. M. de Matos, Isabella de O. F. de Sousa, Letícia C. Ruiz, Leonardo E. de A. Silva, Virmondes Rodrigues, Carlo J. F. de Oliveira, Marcos V. da Silva, Lucas Anhezini, Anielle Christine A. Silva

**Affiliations:** † Strategic Materials Laboratory, Physics Institute, 28112 Federal University of Alagoas, Campus A. C. Simões. Av. Lourival Melo Mota, S/N, Tabuleiro do Martins, Maceió 57072-970, Alagoas, Brazil; ‡ Rede Nordeste de Biotecnologia (RENORBIO), Chemistry Institute, Federal University of Alagoas, Campus A. C. Simões. Av. Lourival Melo Mota, S/N, Tabuleiro do Martins, Maceió 57072-970, Alagoas Brazil; § Laboratory of In Vivo Toxicity Analysis, Institute of Biological Sciences and Health, Federal University of Alagoas, Campus A. C. Simões. Av. Lourival Melo Mota, S/N, Tabuleiro do Martins, Maceió 57072-970, Alagoas, Brazil; ∥ Department of Immunology, Microbiology and Parasitology, Federal University of Triângulo Mineiro, Av. Frei Paulino, no. 30—Bairro Abadia, Uberaba 38025-180, Minas Gerais, Brazil; ⊥ Department of Microbiology, Immunology and Parasitology, Federal University of Triângulo Mineiro, 100 Vigário Carlos St. 7o and 8o floor, Uberaba 38025-350, Minas Gerais, Brazil

## Abstract

This study presents the innovative application of simonkolleite (SM), its nanocomposite with ZnO nanocrystals (NCs), and ZnO nano-/microcrystals to explore their bactericidal and biocompatibility properties for potential biomedical applications. A systematic evaluation of antibacterial activity, antibiofilm efficacy, reactive oxygen species (ROS) production, and Drosophila melanogaster as an in vivo model was performed. The synthesis of SM, SM/ZnO nanocomposites (NCPs), and ZnO nano- and microcrystals was performed at different annealing temperatures: SM at 100 °C, SM/Zn NCPs (63.6% and 69.5% of ZnO NCs) at 250 and 500 °C, respectively, and ZnO NCs and microcrystals (MCs) at 500 and 1000 °C, respectively. Notably, ZnO NCs and MCs have demonstrated pronounced cytotoxicity in the D. melanogaster model, correlating with their ability to rapidly generate reactive oxygen species (ROS), contributing to oxidative stress and cellular damage. The SM/ZnO NCPs (250 °C) exhibited superior biocompatibility, suggesting that the SM partial conversion to ZnO may mitigate toxicity while retaining antimicrobial efficacy. Furthermore, subinhibitory concentrations of SM effectively inhibited biofilm formation, a critical factor in bacterial resistance, highlighting their potential applications in medical device coatings and antimicrobial therapies. Collectively, this research underscores the promising role of these materials in addressing antibiotic resistance and enhancing biocompatibility in biomedical applications.

## Graphical abstract

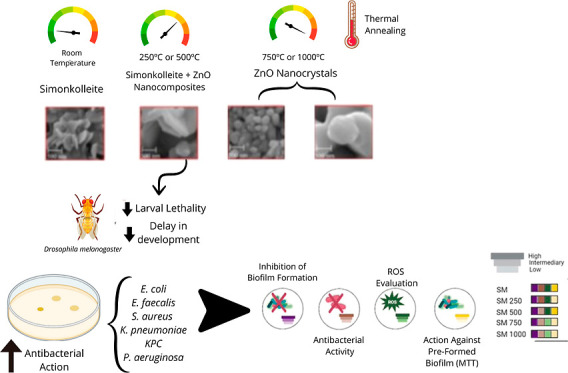

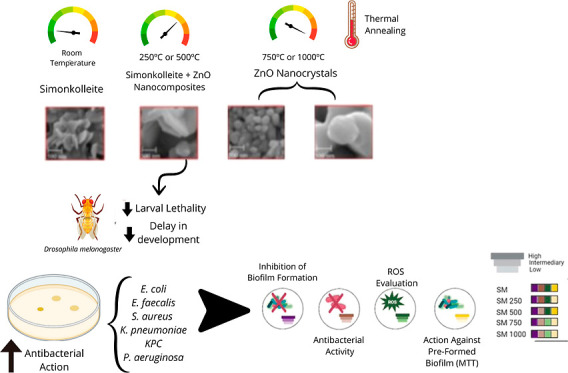

## 1 Introduction

Bacterial infections pose a significant global health threat exacerbated by the growing prevalence of antimicrobial resistance. This issue increases morbidity and mortality rates while complicating effective treatment strategies. For instance, methicillin-resistant Staphylococcus aureus (MRSA) infections result in approximately 11,000 deaths annually in the United States, while Klebsiella pneumoniae carbapenemase (KPC) strains have a global prevalence of 14%.^1–3^ Compounding these challenges, bacterial biofilms form structural defenses that hinder drug penetration and foster resistance, significantly diminishing the efficacy of conventional antibiotics.^1–3^

Nanotechnology offers innovative solutions to these challenges through the development of biocompatible nanomaterials. Zinc oxide (ZnO) nanocrystals, recognized as safe by the Food and Drug Administration (FDA), have demonstrated unique properties, such as producing reactive oxygen species (ROS), which induce oxidative stress in bacteria and cancer cells. These properties make ZnO nanocrystals effective against biofilm-associated infections and antimicrobial resistance.^4,5^ Simonkolleite (SM), a zinc chloride hydroxide monohydrate, also shows promise in biomedical applications due to its electrochemical activity and conductivity.^6,7^ Thermal annealing of SM forms SM/ZnO nanocomposites (NCPs), which enhance cytotoxicity against cancer cells, although studies on their antibacterial and biofilm-preventing capabilities remain limited^6,7^

The fruit fly (Drosophila melanogaster) offers a cost-effective and ethically sound alternative to traditional animal models for studying nanomaterial toxicity and biocompatibility. With a short life cycle, high reproductive capacity, and evolutionary conservation of key physiological mechanisms, Drosophila provides valuable insights into human disease modeling and nanotoxicology. Its use aligns with the “Three Rs” principles endorsed by the European Center for the Validation of Alternative Methods (ECVAM), making it an ideal model for in vivo investigations of nanomaterials.^8,9^

This study investigates the innovative application of SM, its NCPs with ZnO nanocrystals, and ZnO nano-/microcrystals to evaluate their bactericidal and biocompatibility properties. By systematically assessing antibacterial activity, antibiofilm efficacy, ROS production, and in vivo effects using D. melanogaster, this work aims to advance the development of nanomaterials for biomedical applications, addressing critical challenges posed by antimicrobial resistance and biofilm-related infections.

## 2 Material and Methods

### 2.1 Synthesis and Characterization of Simonkolleite and Its Zinc Oxide Transformation

The SM was synthesized using the methodology of Silva et al., and the thermal annealings were performed at 250 °C/1 h and 500 °C/h, forming SM/ZnO NCPs containing the following composition: 40SM/60 and 30SM/70ZnO, respectively.^10^ The thermal annealing at 750 °C/1 h caused the complete transformation of SM into ZnO, forming ZnO NCs, and increasing the temperature to 1000 °C formed ZnO MCs. In addition, the investigation of the optical, structural, morphological, and photocatalytic performances was made by Silva et al.^10^

### 2.2 Bioassays In Vivo

D. melanogaster specimens from the *Canton S* lineage were cultured on a conventional cornmeal medium comprising cornmeal, agar, yeast extract, sugar, and propionic acid. The rearing conditions maintained a temperature of 25 ± 1 °C, a humidity level between 60 and 70%, and a well-defined 12 h light and 12 h dark cycle. A 10 mg/mL stock solution was prepared, diluted in ultrapure water, and subjected to a 30 min sonication process for subsequent use in Drosophila experiments. The solution was then blended with a standard *Drosophila* culture medium at various concentrations. The control group consisted solely of the standard Drosophila culture medium.

The developmental assay was performed to evaluate any developmental effect that SM could have on the overall development of Drosophila. For this, males and females (*n* = 100) were kept in a 6 oz plastic bottle containing a grape juice-based agar cap at the bottom, and the embryo collection was carried out in 8 h intervals. After 24 h of the egg collection, embryos developed into larvae, and these newly hatched larvae were then transferred to experimental vials containing 4 mL of a standard cornmeal media added with the same concentrations of the assays in vitro (0.015, 0.030, 0.06, and 0.120 mg/mL) and standard media as a control. We set up six replicates containing 35 larvae for each concentration, and monitored animals during the following stages of development. We analyzed the pupation daily and determined the pupation rate under each experimental condition. To determine the pupation rate per day (% pupation per day), we calculated the frequency of pupae each day (PD) to the total number of pupae formed (PT). We determined the larval lethality rate by calculating the difference between the total number of L1 larvae (LT) transferred to the experimental vials and the total number of pupae (PT). We expressed it as a percentage [(LT – PT/PT) × 100]. We also calculated the percentage of animals that reached the pupal stage to the number of larvae that initiated the experiment (% total pupation).

### 2.3 Bacterial Strains and Culture

This study used Escherichia coli ATCC 25922, Enterococcus faecalis ATCC 29212, Pseudomonas aeruginosa ATCC 27853, K. pneumoniae ATCC 70063 and their resistant clinical strain, KPC (K. pneumoniae Carbapenemase), and S. aureus ATCC 29213 and their resistant clinical strain, MRSA (methicillin-resistant S. aureus).

Stock cultures were maintained at −80 °C in BHI (brain heart infusion) broth supplemented with 20% (v/v) glycerol. The strains were maintained at 37 °C and cultured overnight before the experiments. They belong to the Federal University of Triângulo Mineiro Laboratory of Immunology—Uberaba/MG, Brazil.

### 2.4 Effect of Materials on Bacterial Growth

The effect of materials on bacterial growth was quantified by broth microdilution. Overnight cultures of all bacterial strains were adjusted to 0.5 McFarland and diluted 1:100 to E. coli, K. pneumoniae, and KPC and 1:10 to P. aeruginosa, E. faecalis, S. aureus, and MRSA. Then, 100 μL of the culture was transferred to a 96-well plate containing 100 μL of BHI broth (control) and 100 μL of NCPs and different thermal treatments of SM-250, SM-500, SM-750, and SM-1000 to final concentrations of 100 μg/mL, 50 μg/mL, 25 μg/mL, 12.5 μg/mL, and 6.25 μg/mL in each well. Plates were incubated for 3 h at 37 °C according to bacterial growth kinetics. The absorbance was measured at 475 nm.

Growth inhibition was presented as the percentage of growth reduction compared with the control condition. The experiment was performed in three replicate wells per microtiter plate for each strain.

### 2.5 Reactive Oxygen Species (ROS) Assay

The production of reactive oxygen species (ROS) was investigated by the fluorescence intensity in a microplate assay. Briefly, 0.5 McFarland’s standards (1.5 × 10^8^ CFU/mL) of adjusted bacteria cells in the logarithmic phase were stained with 2′,7′-dichlorodihydrofluorescein diacetate (DCFH-DA, 0.1 μM) for 60 min, followed by centrifugation (3,500 rpm for 10 min). The supernatant was discarded, and the pellet was resuspended in a BHI broth. After, cells were treated with 100 μg/mL (100 μL) of tested NCPs for 30 min in darkness. The fluorescence was determined at Ex485 and Ex535 wavelengths and expressed in arbitrary fluorescence units (AFU).

### 2.6 Bacterial Antibiofilm Activity of Materials Assay

The inhibitory effect of materials on bacterial biofilms and activity against preformed biofilms were evaluated. For the antibiofilm formation assay, overnight cultures of all bacterial strains were incubated in BHI broth and adjusted to 0.5 McFarland standards. Then, 100 μL of culture was transferred to a 96-well polystyrene microtiter containing 100 μL of fresh BHI broth (control) and BHI containing subinhibitory concentrations of NCPs that showed the inhibitory activity of bacterial growth. After incubation for 24 h at 37 °C, culture media was discarded, and wells were washed three times with saline solution (8.5 mg/mL NaCl) to remove unattached cells. Biofilms were fixed with 200 μL of cold methanol (Synth) for 15 min. Methanol was removed, and the biofilms were dried at room temperature. Then, 100 μL of crystal violet solution (10 mg/mL) was added, and after 15 min, plates were washed 3 times with 200 μL of saline solution. Biofilms were dried at room temperature, and 200 μL of acetic acid solution (330 mg/mL) was added for 20 min to solubilize the crystal violet. The total biofilm biomass was measured by absorbance at 600 nm, and the percentage of inhibition of biofilm formation was expressed by the formula 1 – (OD_treated_/OD_control_) × 100. The experiment was performed in triplicate for each strain, with three independent experiments.

Cells were cultivated, and biofilms were performed without NCPs to analyze the consequence of NCPs on the viability of bacterial preformed biofilms, as described above. After 24 h at 37 °C incubation, unattached cells were removed, and wells were washed 3 times with 200 μL of saline solution. After, the MTT assay was accomplished according to the manufacturer’s directions. After the biofilms were washed, 5 μL of MTT labeling reagent (0.5 mg/mL) was added, and the mixture was incubated at 37 °C for 4 h. Then, 100 μL of solubilization buffer was added, and the mixture was incubated for 24 h at 37 °C. After incubation, absorbance was measured at 550 and 600 nm. The experiment was performed in triplicate for each strain, with three autonomous experiments.

### 2.7 Statistical Analysis

We performed all statistical analyses using GraphPad Prism software (version 8.0, GraphPad Software Inc., San Diego, CA, USA). The pupation rate and larval and pupal lethality data were submitted to analyze the homogeneity of variance with the Shapiro–Wilk test. Parametric data were subjected to a two-way analysis of variance (ANOVA) followed by the Tukey test to compare the mean values. We adopted a significance level of 5%, and the data are shown as the mean ± standard error of the mean (SEM).

## 3 Results

The samples SM (SM100), SM/ZnO NCP (SM250, SM500), ZnO NCs (SM750), and ZnO MCs (SM1000) were carefully characterized to evaluate their structural, thermal, and photocatalytic properties (Supporting Information S1 and S2). Each sample represents a distinct composition: SM100 corresponds to pure simonkolleite, while SM250 (60% ZnO–40% SM) and SM500 (70% ZnO–30% SM) are SM/ZnO NCPs. The SM750 and SM1000 represent ZnO NCs and MCs, respectively. These samples were obtained through thermal annealing at increasing temperatures, progressively transforming SM into ZnO. The photocatalytic analysis showed that SM (SM100) exhibited the lowest degradation efficiency, indicating that simonkolleite is less effective at photocatalytic degradation. On the other hand, ZnO NCs (SM750) and MCs (SM1000) exhibited higher degradation rates, which correlated with the increased production of reactive oxygen species (ROS) under these conditions (Supporting Information S3). The ability of ZnO crystals to generate ROS is a crucial factor in their enhanced photocatalytic performance as it leads to the degradation of organic pollutants.

In vivo experiments were conducted using D. melanogaster larvae, a well-established model organism for toxicity and developmental studies, to assess the biological impact of these materials. The effect concentrations on larval development were examined, focusing on critical stages, such as the transition from larva to pupa. The results revealed that larvae exposed to SM/ZnO NCPs (SM250 and SM500) at a concentration of 0.120 mg/mL experienced a two-day delay in reaching the pupal stage compared to the control group (Figure 1). This developmental delay suggests that these NCPs may interfere with vital physiological processes during the early stages of Drosophila development. Interestingly, the SM in the same concentration did not cause a significant delay in development, thus indicating that the increase in ZnO NCs in the NCPs decreases biocompatibility.

**Figure 1:**
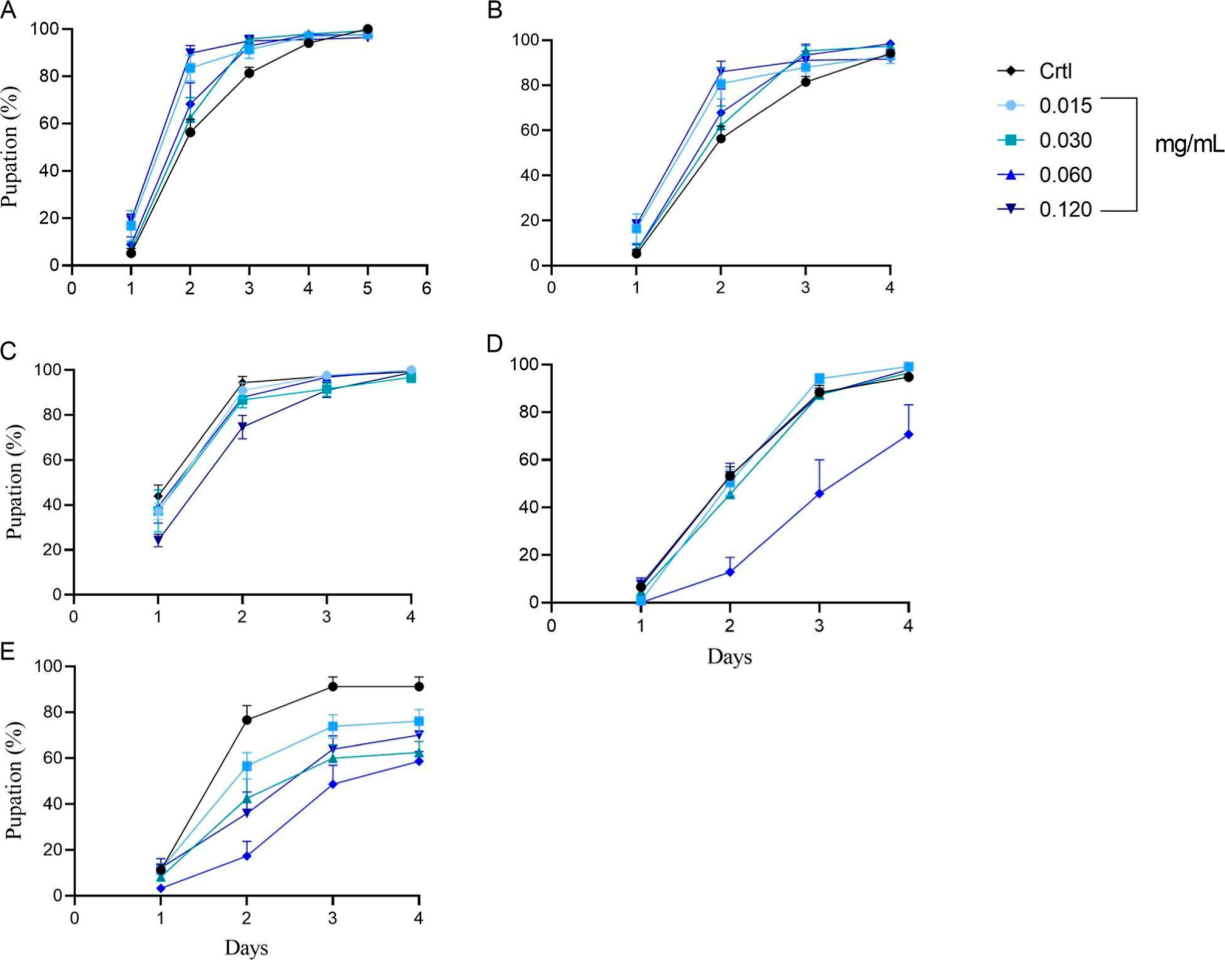
Daily pupation rate of D. melanogaster during exposure in standard culture medium supplemented with (A) SM, (B) SM/ZnO250 NCPs (SM250), (C) SM/ZnO500 NCPs (SM500), (D) ZnO NCs (SM750), and (E) ZnO MCs (SM1000) at concentrations of 0.015, 0.030, 0.060, and 0.120 mg/mL. Data are presented as the mean ± SEM.

At higher concentrations, such as 0.7 and 1.4 mg/mL, a pronounced increase in larval lethality was observed for samples of ZnO NCs (SM750) and ZnO MCs (SM1000), with mortality rates ranging from 80% to 100% (data not shown). This high lethality highlights the toxic effects of ZnO, nano- or microscale, at elevated concentrations, reinforcing the importance of dose when evaluating the biocompatibility of these materials. In contrast, only SM (SM100) and the SM/ZnO NCPs with a low percentage of ZnO NCs (SM250) demonstrated lower toxicity. Thus, SM250 is the most biocompatible sample.

Furthermore, larvae exposed to SM500, SM750, and SM1000 across all concentrations tested exhibited significant larval lethality rates between 30% and 40% compared with the control (Figure 2 A). These increased lethality rates corresponded to a decrease in the total pupation rate for ZnO NCs (SM750) and ZnO MCs (SM1000) (Figure 2 B), suggesting that ZnO induces physiological stress that impacts survival and developmental progression. By contrast, larvae exposed to SM/ZnO NCPs (SM250) showed no significant difference in survival and pupation rates compared to the control group, further supporting the lower toxicity of these NCPs. Thus, the synergism of SM decreases the toxicity of ZnO NCs.

**Figure 2:**
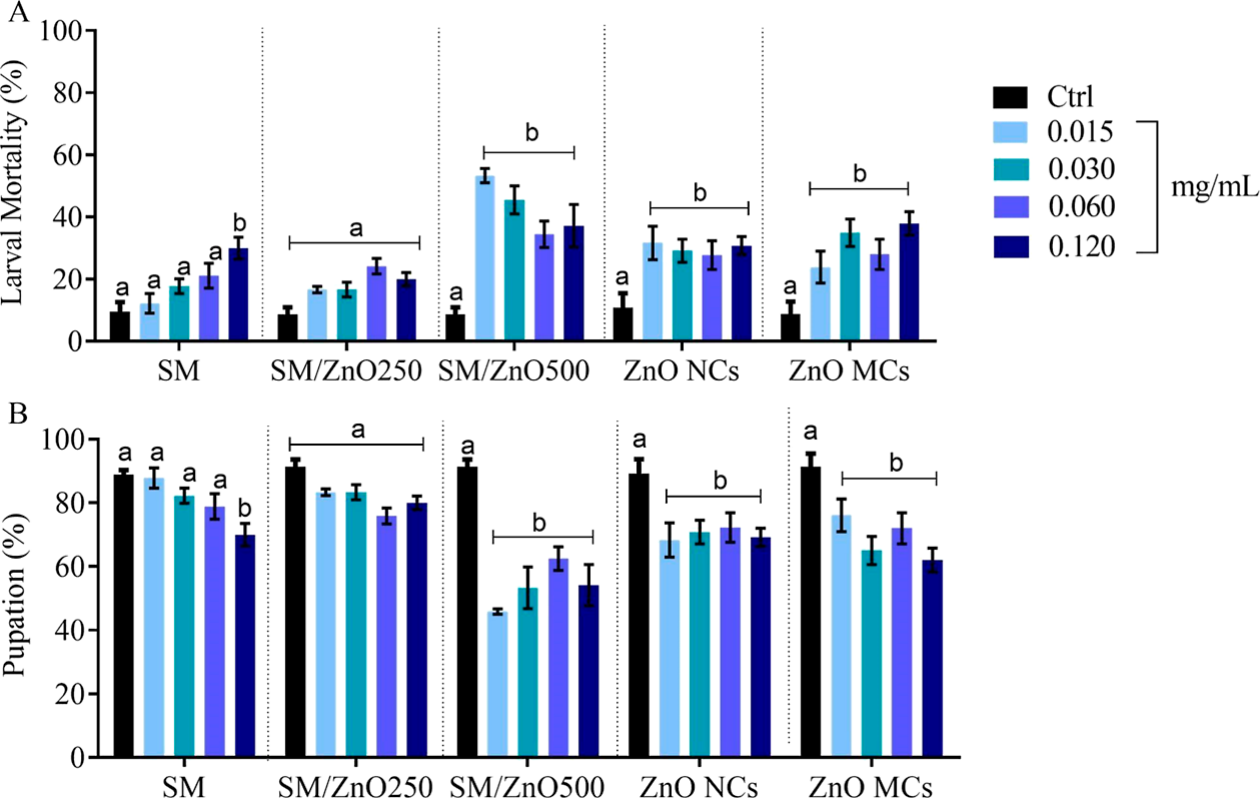
Larval mortality (A) and pupation rates (B) in Drosophila melanogaster during exposure in standard Drosophila culture medium added with SM (SM100), SM/ZnO NCPs (SM 250), SM/ZnO500 NCPs (SM500), ZnO NCs (SM750), and ZnO MCs (SM1000) at concentrations of 0.015, 0.030, 0.060, and 0.120 mg/mL. a and b represent the statistical difference between groups within the same concentration. The data is presented as the mean ± SEM.

The observed differences in toxicity among the samples can be attributed to the synergistic effects of the ZnO NCs and SM in the NCPs. While ZnO NCs (SM750) and ZnO MCs (SM1000) exhibit more ROS production and higher toxicity, SM and SM/ZnO NCPs (SM250) appear to mitigate these effects, resulting in better biocompatibility. This result highlights the importance of the material composition in determining the biological interactions of nanomaterials. Among the tested samples, SM250 exhibited the lowest lethality rate (*p* = 0.0001) compared to SM/ZnO500 NCs, SM750, and SM1000 (Supporting Information S4). Therefore, SM/ZnO NCPs are the most promising candidate for biomedical applications where biocompatibility is crucial.

In addition, the SM/ZnO NCPs (SM250) are the sample with the best photocatalytic performance and biocompatibility. This finding underscores the potential of SM/ZnO NCPs as materials for applications where antibacterial activity and low toxicity are required, such as in medical devices or environmental remediation technologies.

All tested samples could inhibit bacterial growth across all examined strains, as illustrated in Figure 3. At the highest concentrations (100 and 50 μg/mL), both SM and SM/ZnO NCP (SM250) showed significant inhibitory effects. The synergism of SM with the ZnO NCs forming the SM/ZnO NCP (SM250) improved the inhibition rate, exceeding 89% against E. coli. At the same time, SM displayed an inhibition range of 92%–100% against E. faecalis (Figure 3A,B). This highlights the potent antibacterial properties of these nanomaterials at higher doses.

**Figure 3:**
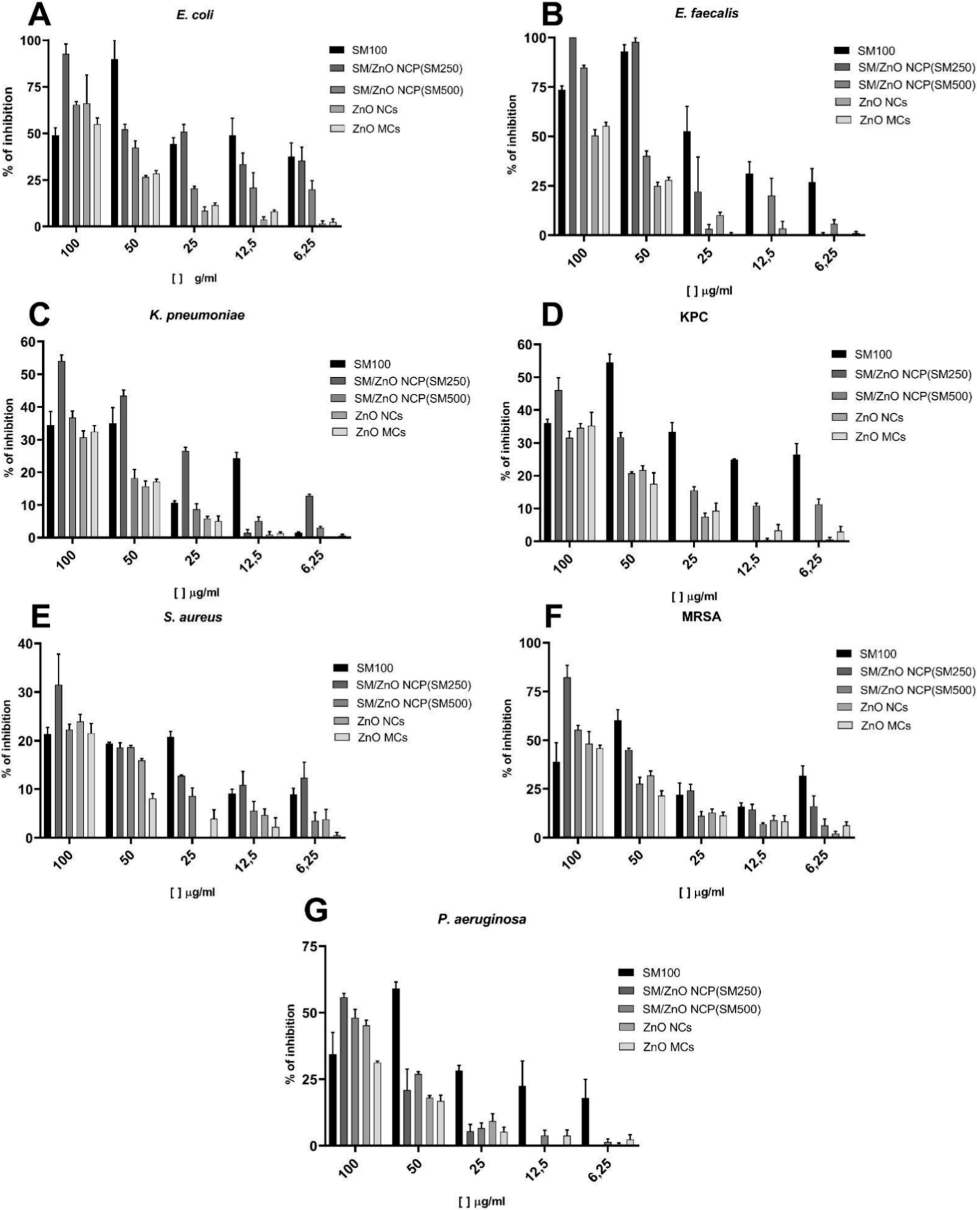
Percentage of bacterial growth inhibition by the microdilution method to SM (SM100), SM/ZnO NCP (SM250), SM/ZnO NCP (SM500), ZnO NCs (SM750), and ZnO MCs (SM1000): (A) E. coli, (B) E. faecalis, (C) K. pneumoniae, (D) KPC; (E) S. aureus, (F) MRSA, and (G) P. aeruginosa. Absorbance was measured at 475 nm, and the percentage was relativized with nontreated conditions.

The SM showed a 54.4% inhibitory effect against the carbapenem-resistant K. pneumoniae (KPC) strain. In comparison, the SM/ZnO NCP (SM250) at a concentration of 100 μg/mL inhibited 82.5% of the methicillin-resistant S. aureus (MRSA) strain (Figure 3D,F). These results indicate that SM presents better antimicrobial activity than ZnO nano-/microparticles.

The superior antibacterial efficacy of SM/ZnO NCPS, particularly sample SM250, suggests that these materials could be highly effective in addressing challenging bacterial strains, including multidrug-resistant pathogens. The data reinforce the potential of SM-based nanomaterials as promising candidates for antimicrobial applications, offering an alternative approach to combating bacterial infections.

The highest level of antibacterial activity varied across the different materials tested, and no direct correlation was observed between the compound concentration and antibacterial action. The final concentrations for wells are expressed in Table 1.

**Table 1:** Sample Concentrations against Bacteria of Clinical Relevance Used in Assay Antibiofilms

	nanomaterial concentration (μg/mL)				
bacterial strain	SM SM100	SM/ZnO NCP SM250	SM/ZnO NCP SM500	ZnO NCs SM750	ZnO MCs SM1000
E. coli				25.0	25.0
E. faecalis		12.5	25.0	12.5	25.0
P. aeruginosa		12.5	12.5	12.5	12.5
K. pneumoniae	6.25	12.5	12.5	25.0	25.0
KPC		25.0		12.5	12.5
S. aureus			6.25	25.0	6.25
MRSA			6.25	6.25	6.25

The method of fluorescence intensity was employed to assess the production of ROS in the bacterial strains, as shown in Figure 4. The analysis revealed that simonkolleite effectively enhanced ROS production in E. coli, K. pneumoniae, S. aureus, and methicillin-resistant S. aureus (MRSA). Among the samples tested, the SM/ZnO NCPs (SM250) stood out by significantly boosting ROS production in E. coli, S. aureus, and MRSA. This indicates that the sample has a strong capacity to induce oxidative stress, which plays a key role in its bactericidal activity.

**Figure 4:**
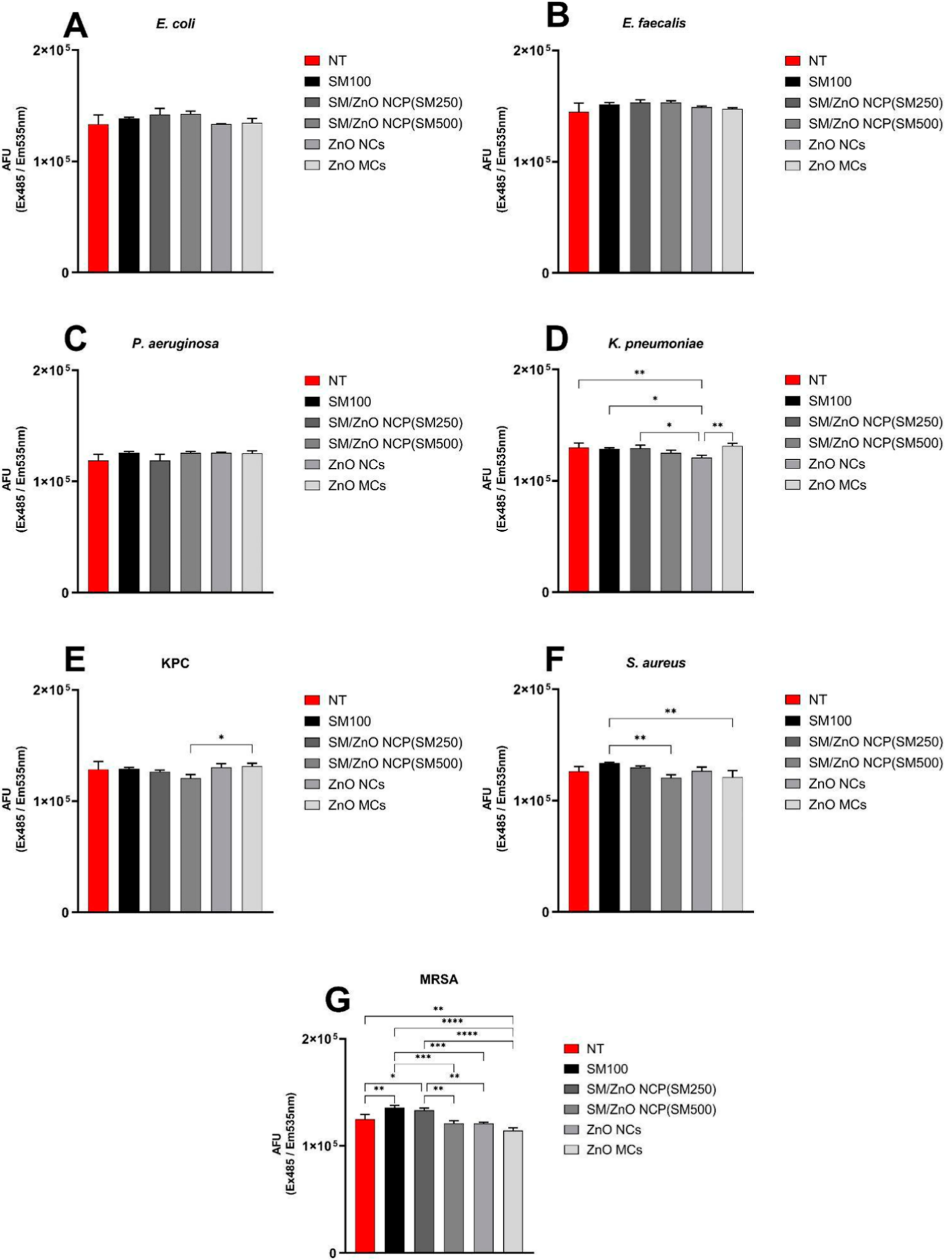
Reactive oxygen species (ROS) production in (A) E. coli, (B) E. faecalis, (C) P. aeruginosa, (D) K. pneumoniae, (E) KPC, (F) S. aureus, (G) MRSA, and bacteria treated with o SM (SM100), SM/ZnO NCPs (SM250), SM/ZnO NCP (SM500), ZnO NCs (SM750), and ZnO MCs (SM1000) and no treatment (NT). The fluorescence was determined at Ex485/Ex535 wavelengths and expressed in arbitrary fluorescence units (AFU).

In contrast, increasing the percentage of ZnO NCs in the SM/ZnO NCPs (SM500) had a more variable effect on the ROS production. It enhanced ROS generation in E. coli but reduced ROS levels in K. pneumoniae, carbapenem-resistant K. pneumoniae (KPC), S. aureus, and MRSA. This reduction in ROS production suggests that the specific composition of SM/ZnO NCP (SM-500) may selectively influence bacterial strains, affecting their susceptibility to oxidative stress.

The ZnO NCs (SM750) exhibited a greater capacity for inducing ROS production, particularly in E. coli, KPC, and S. aureus. The higher levels of ROS generated by this sample may contribute to its more potent antibacterial effects against these strains. On the other hand, ZnO MCs (SM1000) demonstrated a varied response. It enhanced ROS production in E. coli, K. pneumoniae, and KPC. Still, it resulted in lower ROS levels in S. aureus and MRSA, indicating that its effectiveness may vary depending on the bacterial species, in excellent agreement when we analyze the ROS production generated in the photocatalysis experiments.

No significant differences in ROS production were observed for E. faecalis and P. aeruginosa across the tested samples. These results suggest that the ROS-inducing potential of simonkolleite and its derivatives is strain-dependent, displaying a greater efficacy against specific bacteria. The ability to modulate ROS production provides valuable insights into their mechanisms of action and their potential as antibacterial agents.

Subinhibitory concentrations of the compounds displaying antimicrobial activity were employed to evaluate the impact of the synthesized materials on biofilm formation. All bacterial strains tested exhibited the capacity to form biofilms, which allowed for a comprehensive assessment of the materials’ inhibitory effects on biofilm formation (Figure 5). The results demonstrate that ZnO NCs (SM750) and ZnO MCs (SM1000) were particularly effective, showing 75.1% and 66.2% biofilm inhibition rates against K. pneumoniae (Figure 5C,D).

**Figure 5:**
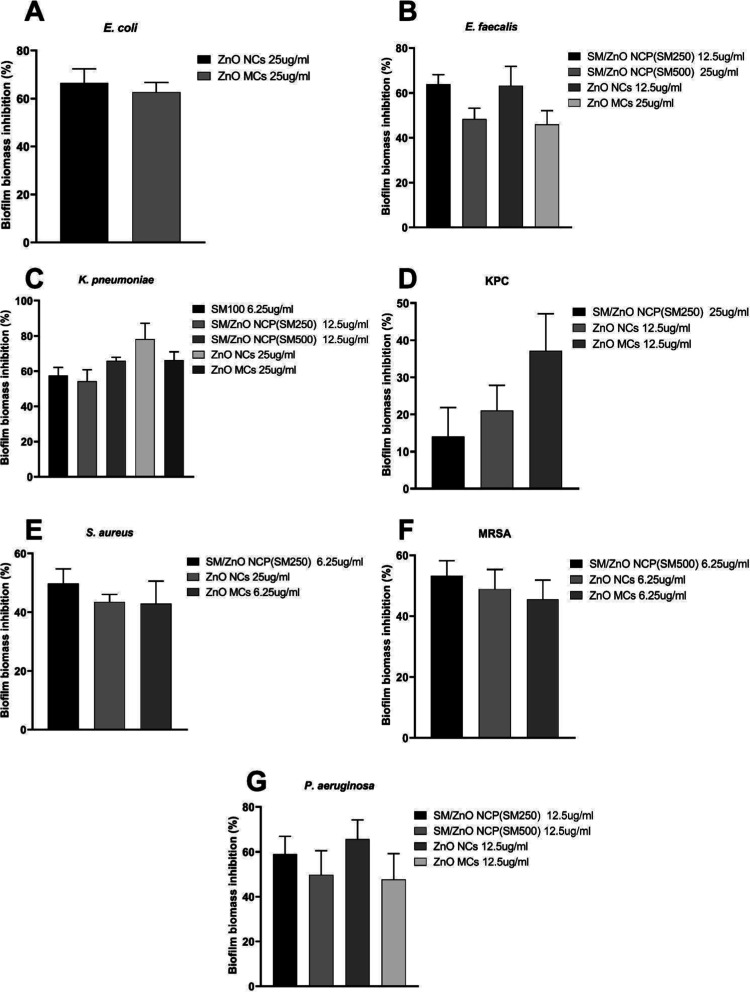
Percentage of biofilm biomass (crystal violet) inhibition in (A) E. coli, (B) E. faecalis, (C) K. pneumoniae, (D) KPC, (E) S. aureus, (F) MRSA, and (G) P. aeruginosa by treatment with subinhibitory concentrations of SM (SM100), SM/ZnO NCP (SM250), SM/ZnO NCPs (SM500), ZnO NCs (SM750), and ZnO MCs (SM1000). The absorbance was measured at 600 nm, and the percentage was relativized to nontreated conditions.

Furthermore, these ZnO nano-/microcrystals also displayed significant inhibitory activity against biofilms formed by methicillin-resistant S. aureus (MRSA), with inhibition rates exceeding 40%. Given that MRSA strains resist conventional antimicrobial agents, these findings underscore the potential alternative approaches for preventing biofilm formation in hard-to-treat bacterial strains.

The ability of these materials to inhibit biofilm formation at subinhibitory concentrations highlights their potential as antibacterial agents and as practical tools for disrupting biofilm-related bacterial infections. Due to the protective nature of biofilms, these infections are notoriously difficult to treat. This property could offer significant advantages in clinical settings where biofilm-associated infections contribute to increased resistance and treatment failure.

The ability of SM to reduce bacterial viability in the biofilm state was evaluated by using the MTT assay (Figure 6). Preformed biofilms treated with SM and its NCPs with ZnO NCs demonstrated a notable capacity to inhibit biofilm formation across all tested bacterial strains except E. faecalis (data not shown). This suggests that the presence of SM exhibits broad-spectrum biofilm inhibition.

**Figure 6:**
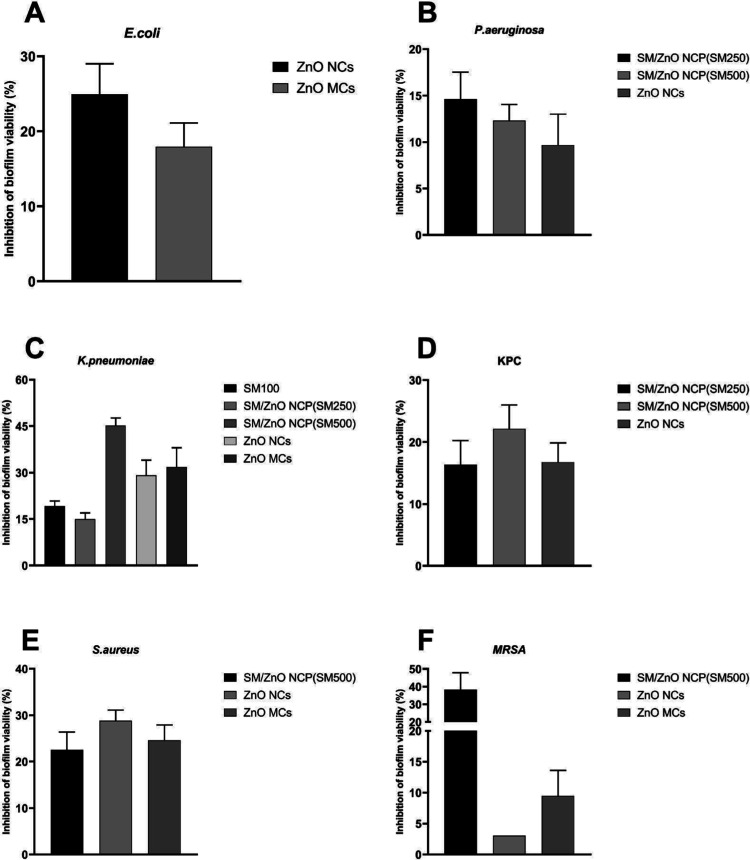
Percentage inhibition viability (MTT) of preformed biofilms of (A) E. coli, (B) P. aeruginosa, (C) K. pneumoniae, (D) KPC, (E) S. aureus, and (F) MRSA treated with subinhibitory concentrations of SM (SM100), SM/ZnO NCP (SM250), SM/ZnO NCP (SM500), ZnO NCs (SM750), and ZnO MCs (SM1000). The absorbance was measured at 550–600 nm, and the percentage was relativized with nontreated conditions.

The increase of ZnO NCs in the SM/ZnO NCPs (SM500) exhibited a significant reduction in biofilm viability, inhibiting over 40% of the biofilm formed by K. pneumoniae *.* Furthermore, it showed intense inhibitory action against biofilms produced by methicillin-resistant S. aureus (MRSA), with a nearly 40% reduction in biofilm viability. These results emphasize that increasing ZnO NCs in the SM/ZnO NCPs (SM500) effectively disrupts biofilm viability in Gram-negative and Gram-positive pathogens, including multidrug-resistant strains. Thus, the ability of SM/ZnO NCPs (SM500) to minimize biofilm viability highlights its potential as a promising material for targeting biofilm-associated infections, which are often resistant to conventional therapies. Significantly reducing biofilm viability helps address one of the key challenges in treating persistent bacterial infections, offering a new avenue for antimicrobial strategies.

## 4 Discussion

The group has demonstrated that SM, SM/ZnO NCP, ZnO NCs, and ZnO MCs are active in tumor cells and seed treatment and control of tomato bacteria.^7,10,11^ Thus, in this work, each sample’s in vivo biocompatibility and antimicrobial activities were investigated, directing the best medical applications of each sample.

Thermal annealing favors the transformation of SM to ZnO, allowing the control of the percentage of ZnO present in each NCP formed. In addition, a temperature of 750 °C favors the complete transformation of SM into ZnO NCs, and temperatures above this favor crystallinity and size, with 1000 °C already forming ZnO microcrystals. Thus, 100 °C forms SM, 250 °C forms SM/ZnO NCP, and increasing to 500 °C increases the percentage of ZnO NCs in the NCP, 750 °C forms ZnO NCs, and 1000 °C forms ZnO MCs.

The lower degradation rate observed for SM is likely due to its wide bandgap (∼3.33 eV), which reduces its susceptibility to photodegradation by limiting the excitation of electrons to the conduction band under typical environmental conditions. This broad bandgap effectively minimizes the generation of reactive electron–hole pairs, key drivers of photochemical reactions, enhancing its structural stability and prolonging its functional lifespan in applications. The SM structures tend to aggregate along the normal direction during growth. In this context, electrons oxidize atmospheric oxygen, forming superoxide radicals (^•^O_2_^–^), which are involved in the oxidation process that degrades organic dyes. The enhanced photocatalytic activity is due to the formation of ZnO and increased size and crystallinity. The rapid generation of hydroxyl radicals (^•^OH) and ^•^O_2_^–^ species is critical for facilitating the photocatalytic process.

Biocompatibility assays using the D. melanogaster model were performed to evaluate the toxicity of SM, SM/ZnO NCPs, ZnO NCs, and ZnO MCs. The results confirmed that the exposure of larvae to SM, ZnO NCs (SM750), and ZnO MCs (SM1000) likely triggered physiological changes, leading to developmental delays, increased mortality, and consequently reduced pupation rates. The formation of ROS leading to redox imbalance can cause oxidative stress, potentially resulting in cellular damage by oxidizing proteins, lipids, and DNA. This process may also disrupt the synthesis of ecdysone, which is critical for regulating molting during the development of the fruit fly.

The ZnO nano- and microcrystals exhibited significant toxicity, leading to developmental delays and larval lethality. Thus, the cellular toxicity is attributable to the nanoparticles’ rapid generation of reactive species, such as ^•^O_2_^–^ and peroxynitrite, as demonstrated in the methylene blue experiments conducted in this study.

Oxidative stress is a key mechanism underlying ZnO toxicity, resulting in alterations in antioxidant synthesis and the deregulation of various signaling pathways, often culminating in cell death. Furthermore, redox imbalance is implicated in neurological disorders, adversely influencing fetal development and ecdysone biosynthesis mechanisms. Ecdysone plays a crucial role in regulating molting and metamorphic transitions in insects, and its synthesis involves a series of oxidation–reduction reactions. A vital factor for the synthesis and functionality of this hormone is the protein glutathione (GSH), along with levels of nitric oxide (NO^•^).^12,13^

The ZnO nanomicrocrystals are more toxic than SM and SM/ZnO NCPs, resulting in developmental delays and increased lethality in D. melanogaster, likely due to redox imbalance. This phenomenon is commonly observed in nanotoxicology studies involving fruit flies. Our group reported it in previous works using pure ZnO NCs or ZnO doped with transition metals at high concentrations.^14–16^

The presence of SM in the SM/ZnO NCPs (SM250) decreases toxicity and has superior biocompatibility of SM. Compared to other treatments, the reduced larval lethality and developmental impact may be attributed to its lower production of reactive species. This suggests that combining SM with ZnO may mitigate toxicity in vivo. However, further investigations are necessary to elucidate the mechanisms underlying the enhanced biocompatibility.

ZnO is considered a safe and nontoxic human-use material, as indicated by the FDA.^17^ It has various potential applications when incorporated into different matrices, providing properties such as UV-A and UV-B ray absorption, antimicrobial activity, and enhanced packaging characteristics, including mechanical strength, stability, and barrier effectiveness.^18–20^ While the antimicrobial properties of ZnO have been documented, our study demonstrates that SM and SM/ZnO NCPs (SM250) exhibit pronounced inhibition of bacterial growth against E. coli, E. faecalis, MRSA, and KPC strains. All of the tested materials displayed antibacterial effects against the examined strains. Infections caused by MRSA and KPC present significant economic and logistical challenges, resulting in high mortality and morbidity rates, and are recognized as urgent public health threats by the CDC.^21,22^

The observed antibacterial activity in the samples with SM, at least in part, can be linked to the production of ROS upon contact with E. coli and MRSA. However, the inhibition of KPC growth might be attributable to alternative antibacterial mechanisms. ROS interact with bacterial cell walls, membranes, DNA, RNA, lipids, and proteins, enhancing cell viability and inducing cell death.^23^ Furthermore, the production of ROS can improve the efficacy of antibiotic treatments.^23^ Additionally, the small size, structure, and crystallinity of NCs may influence their cellular penetration, distribution, and genotoxicity.^24–27^ The multifactorial targets of nanoparticles within bacterial cells complicate resistance acquisition, necessitating multiple genetic mutations to detoxify intracellular and extracellular environments.^28^ This suggests that SM and SM/ZnO NCPs may hold promise for combating MRSA and KPC strains in human health applications.

Bacteria’s ability to grow in biofilms provides a protective niche, and the conformational arrangement of bacterial cells hinders the infiltration of antimicrobial agents, rendering these communities less susceptible to antibiotic therapies.^29^ Moreover, the adhesion capacity of biofilm-forming strains to medical device surfaces poses a significant concern in clinical settings, particularly for immunocompromised individuals.

Our study demonstrated that subinhibitory concentrations of materials effectively inhibited biofilm formation in relevant bacterial strains. We observed over 50% inhibition of biofilm formation by MRSA and KPC using lower SM100, ZnO NCs, and SM/ZnO NPS (SM500) concentrations. While SM500, ZnO NCs, and ZnO MCs exhibited lower growth inhibition, SM and SM250 demonstrated promising antibiofilm activity against MRSA and KPC strains. Furthermore, the presence of SM enhanced antibacterial activity against preformed biofilms. Given that cells in biofilm states exhibit resistance mechanisms to antibiotics, understanding the actions of SM and its thermal variants, which show lower toxicity, may open new avenues for applying these compounds to protect medical device surfaces and effectively eliminate biofilms.

## 5 Conclusion

In conclusion, this study highlights the multifaceted properties of SM, its SM/ZnO NCPs, and ZnO nano-/microcrystals. Their ability to generate reactive oxygen species (ROS) and inhibit biofilm formation establishes them as promising candidates for developing innovative strategies to combat antibiotic resistance. The enhanced photocatalytic activity, combined with their variable biocompatibility profiles, emphasizes the need for further exploration of the mechanisms underlying their biological interactions. Our findings advance our understanding of the synergistic effects between SM and ZnO NCs forming NCPs, demonstrating their potential to enhance antimicrobial efficacy while improving biocompatibility. Consequently, SM, SM/ZnO NCPs, or ZnO nano-/microcrystals may be selected based on specific biomedical applications. This work affirms these materials’ antimicrobial efficacy and toxicological properties, paving the way for their broader utilization in medical contexts.

## Supplementary Material



## Notes

XRD patterns of the samples after synthesis and thermal annealing, all of the nanomaterials’ SEM, and images showing the results of the photocatalytic degradation and linear regression of larval lethality rate among different nanoparticles (PDF)
