# Supplementary material for: From Nanocrystals and Nanocomposites to Microcrystals: The Role of Simonkolleite/ZnO in Overcoming Bacterial Resistance and Ensuring Biocompatibility
Source: ACS Omega. 2025 May 15;10(20):20117–28. doi: 10.1021/acsomega.4c09594 (PMC12120594; doi:10.1021/acsomega.4c09594)
Supplement: Supplementary file 1 [file ao4c09594_si_001.pdf]

From Nanocrystals, Nanocomposites to Microcrystals: The Role of Simonkolleite/ZnO in Overcoming Bacterial Resistance and Ensuring Biocompatibility

Jerusa M. de Oliveira[a-c†#], Maria P. C. Costa [a], Hugo F. Perini[d†], Rafael O. Trevisan[d†], Larissa I. M. de Almeida[a,c], Samanta L. M. de Matos[a,c], Isabella de O. F. de Sousa[d], Letícia C. Ruiz[d], Leonardo E. de A. e Silva[f], Virmondes Rodrigues Jr.[d], Carlo J. F. de Oliveira[d], Marcos V. da Silva[d], Lucas Anhezini[c†], Anielle Christine A. Silva[a,b#]

[a] *Strategic Materials Laboratory, Physics Institute, Federal University of Alagoas, Maceió, Alagoas, Brazil.*
[b] *Rede Nordeste de Biotecnologia (RENORBIO), Chemistry Institute, Federal University of Alagoas, Maceió, Alagoas Brazil.*
[c] *Laboratory of in vivo Toxicity Analysis, Institute of Biological Sciences and Health, Federal University of Alagoas, Maceió, Alagoas, Brazil.*
[d] *Department of Immunology, Microbiology and Parasitology, Federal University of Triângulo Mineiro, Uberaba, Minas Gerais, Brazil.*
[f] *Mycology Laboratory, Institute of Health Sciences, Federal University of Triângulo Mineiro, Uberaba, Minas Gerais, Brazil.*

[#]corresponding authors: Anielle Christine Almeida Silva: aniellechristineas@gmail.com

Jerusa Maria de Oliveira: jerusa.oliveira@fis.ufal.br; oliveira.jerusam@gmail.com

[†] These authors contributed equally to this manuscript.

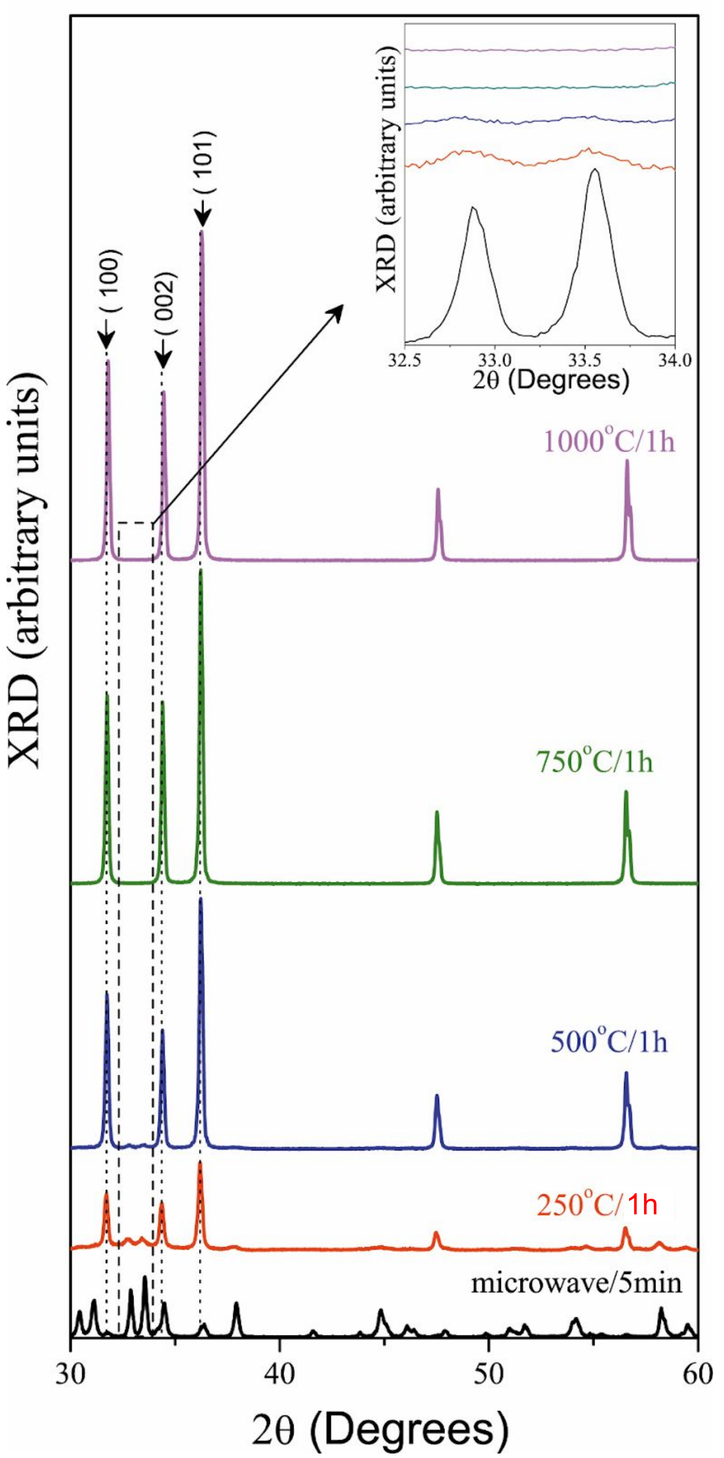

Supplementary 1. XRD patterns of the samples after synthesis and thermal annealing at 250°C/1h, 500°C/1h, 750°C/1h, and 1000°C/1h.

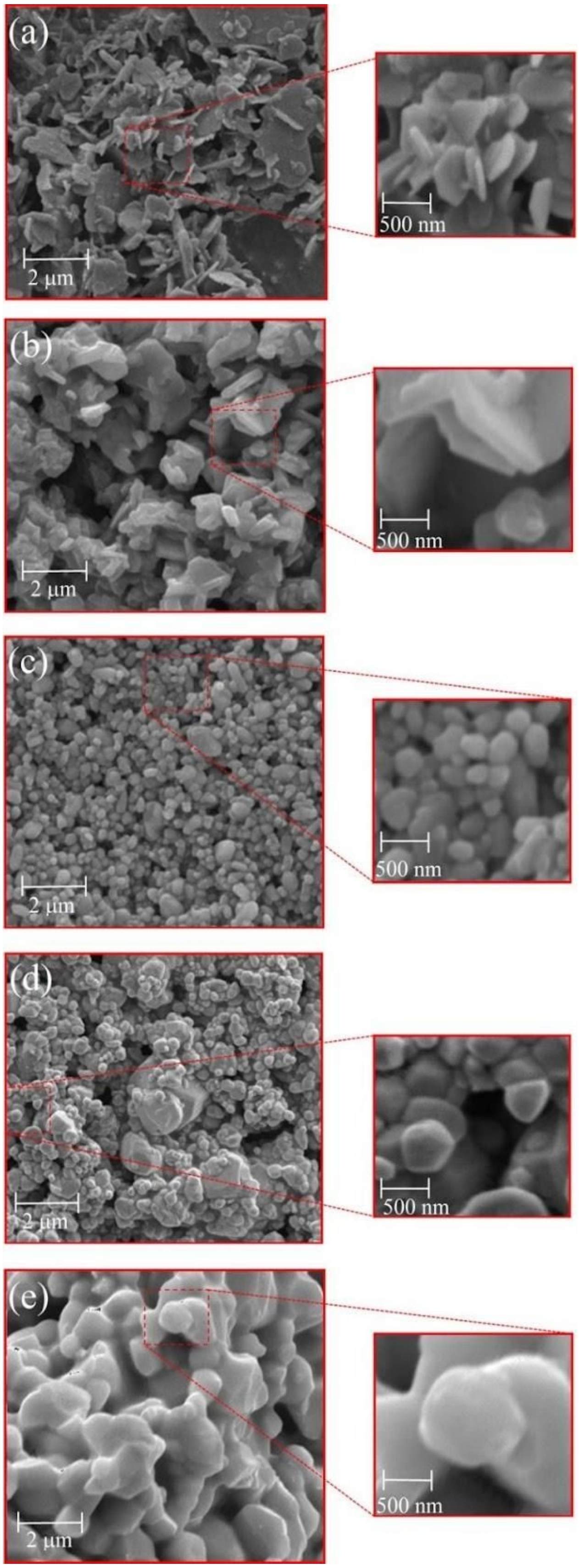

Supplementary 2. The SEM images of (a) SM NCs, (b) 250°C/1h, (c) 500°C/1h, (d) 750°C/1h, and (e) 1000°C/1h.

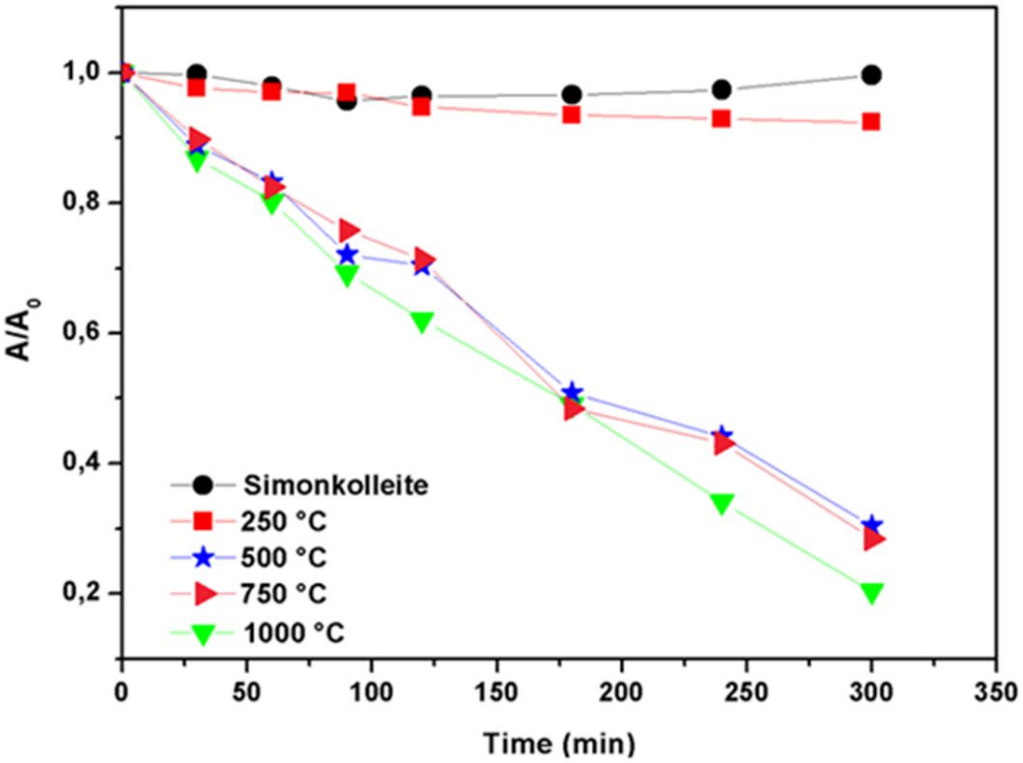

Supplementary 3. Photocatalytic degradation of MB solution by SM and thermal annealing at 250ºC, 500ºC, 750ºC, and 1000ºC.

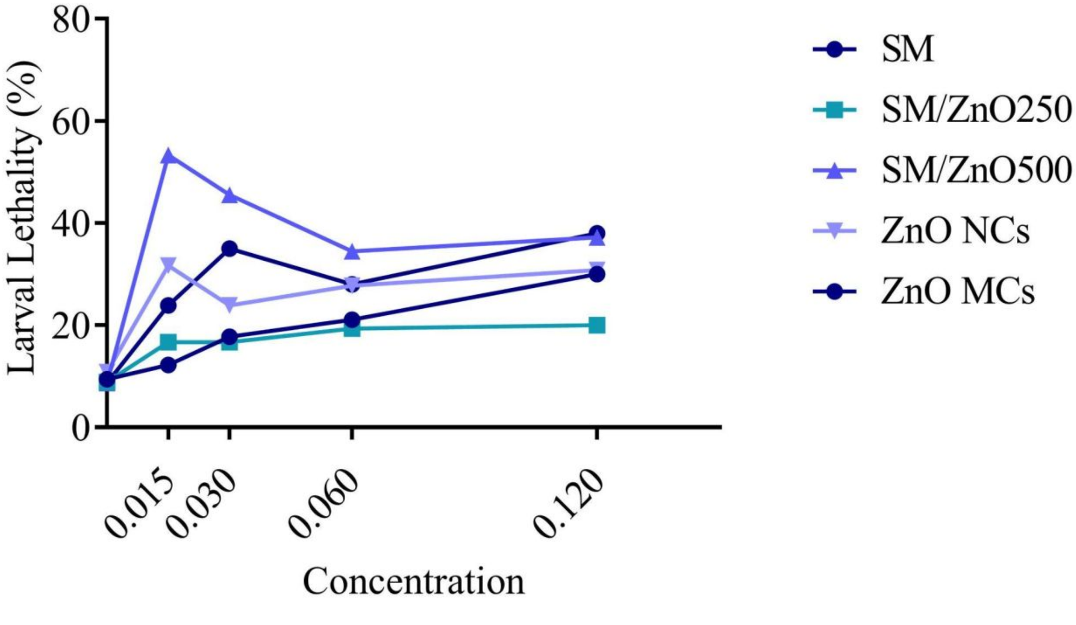

Supplementary 4: Larval lethality rate among different nanoparticles. Simonkolleite after different heat treatments: 250ºC (SM/ZnO250 nanocomposite (NCPs)) demonstrates the lowest rate of larval lethality among the types of nanocomposites of Simonkolleite (SM) and 500ºC (SM/ZnO500) NCPs, 750ºC (ZnO nanocrystals (MCs)), 1000ºC (ZnO MCs) at concentrations of 0.015, 0.030, 0.060, 0.120 mg/mL. The data is presented as mean ± SEM.
